# Occipital spikes of the blind: Insights from EEG source localization

**DOI:** 10.1002/epd2.70231

**Published:** 2026-03-24

**Authors:** Agilda Dema, Douglas Nordli

**Affiliations:** ^1^ Department of Pediatric Neurology University of Chicago Chicago Illinois USA

**Keywords:** cortical visual impairment, EEG source localization, occipital spikes of the blind

“Occipital spikes of the blind” are a rare but striking EEG finding classically observed in individuals with blindness or severe visual impairment.^1^ Despite their sharp occipital morphology, their physiologic significance has remained uncertain, and they are often misclassified as epileptiform. We identified this pattern in a 4‐year‐old girl with cortical visual impairment from occipital encephalocele and applied modern EEG source localization to delineate its cortical origin. Averaged epochs revealed highly stereotyped occipital transients with surface positivity maximal at O1–O2 and a confined field distribution. Dipole modeling localized activity within the primary visual cortex with a confined posterior field and consistent cortical source.

In this case, the combined scalp topography and source model support the interpretation that the observed discharges may represent non‐epileptic, lesion‐associated cortical hyperexcitability and/or adaptive reorganization following visual deafferentation. Recognition of this deceptive pattern may help avoid misclassification during presurgical evaluation for suspected occipital lobe epilepsy and underscores the importance of integrating clinical context, scalp topography, and source analysis in EEG interpretation. However, one case cannot determine whether similar sharply contoured occipital discharges in other blind children behave the same way or remain uniformly benign over time. Therefore, further investigation of additional patients with comparable findings, along with longitudinal follow‐up into adolescence and adulthood, will be important to clarify the long‐term clinical significance and relative benignity of such activity.

More broadly, this case highlights the continuing need for expert oversight when distinguishing physiologic from epileptiform waveforms, particularly as AI‐based spike detection and automated EEG interpretation become more widely adopted (Figure 1).

**FIGURE 1:**
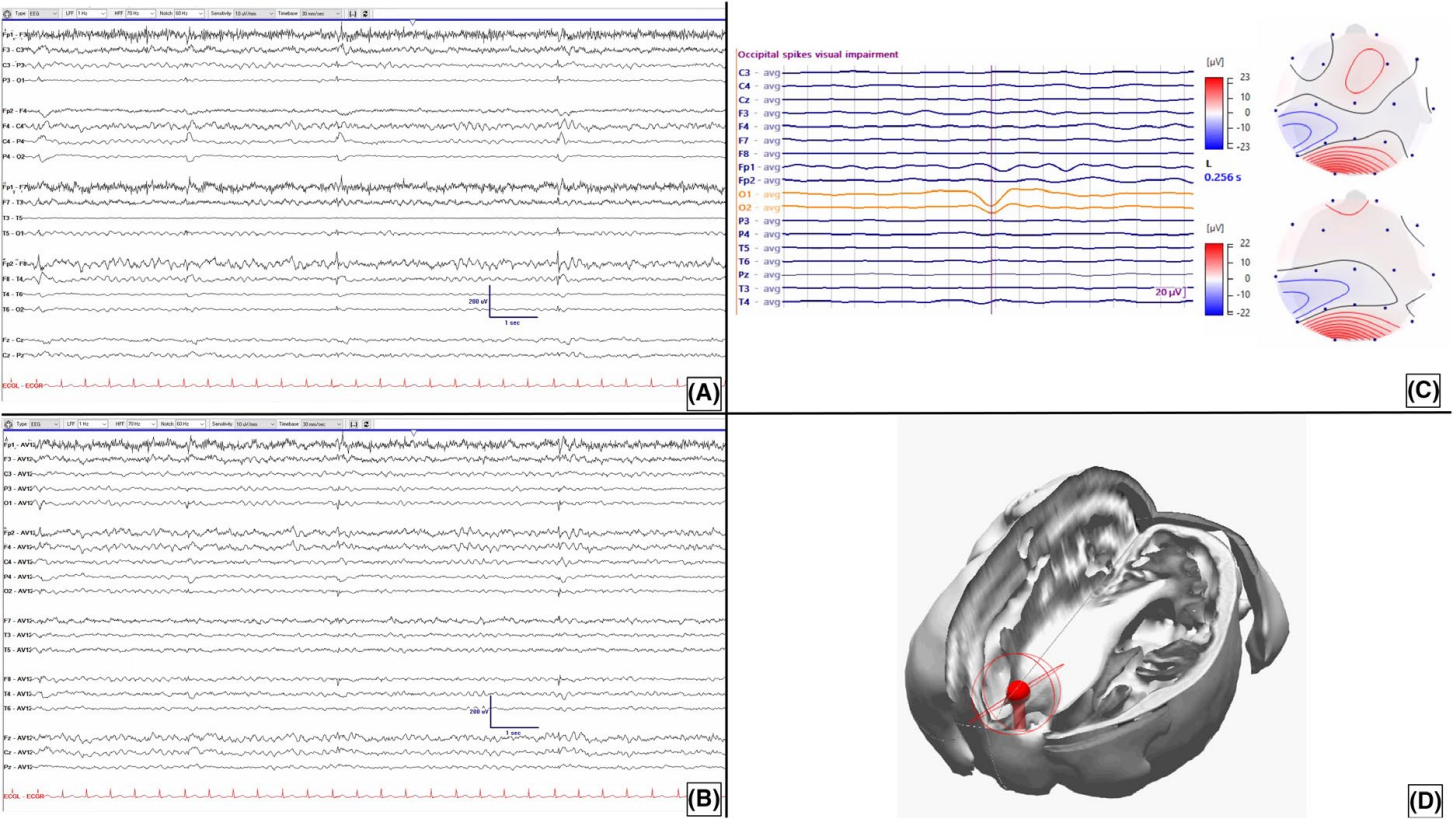
Representative EEG epochs in longitudinal bipolar and average reference montages show sharply contoured occipital transients maximal at O1‐O2 with limited field distribution and no propagation (A, B). Averaged waveform and topographic voltage maps demonstrate posterior positivity with symmetric occipital field confinement (C). Single dipole moving source analysis localizes the origin to the calcarine cortex, consistent with activation of the primary visual area (D).

## FUNDING INFORMATION

The authors received no financial support for the research, authorship, and/or publication of this article.

## CONFLICT OF INTEREST STATEMENT

The authors declare no conflict of interest.

## INFORMED CONSENT

Written informed consent was obtained from the patient's legal guardians for inclusion in this report. All identifying details have been omitted or anonymized to protect patient confidentiality.

**Test yourself**

1. Which of the following best describes the cortical source of “occipital spikes of the blind”?

- Mesial temporal cortex
- Parietal association cortex
- Primary visual (calcarine) cortex
- Frontal eye fields

2. Which EEG characteristic most helps distinguish “occipital spikes of the blind” from epileptiform occipital spikes?

- Sharp morphology
- Surface positivity maximal at O1–O2
- Source localization to primary visual cortex with a confined posterior field
- Increased frequency during sleep

3. What is a likely underlying mechanism for occipital spikes of the blind?

- Deafferentation‐related cortical hyperexcitability in visual cortex
- Secondary epileptogenesis from temporal lobe foci
- Retinal‐originated photic after discharges
- Artifact from eye movement

*Answers may be found in the* *supporting information*.

## Supporting information


Data S1:

Data S2:

## Data Availability

The data that support the findings of this study are available from the corresponding author upon reasonable request.
