# Supplementary material for: Occipital spikes of the blind: Insights from EEG source localization
Source: Epileptic Disord. 2026 Mar 24;28(3):930–2. doi: 10.1002/epd2.70231 (PMC13276702; doi:10.1002/epd2.70231)
Supplement: Supplementary file 1 — Data S1: [file EPD2-28-930-s001.pptx]

## Slide 1
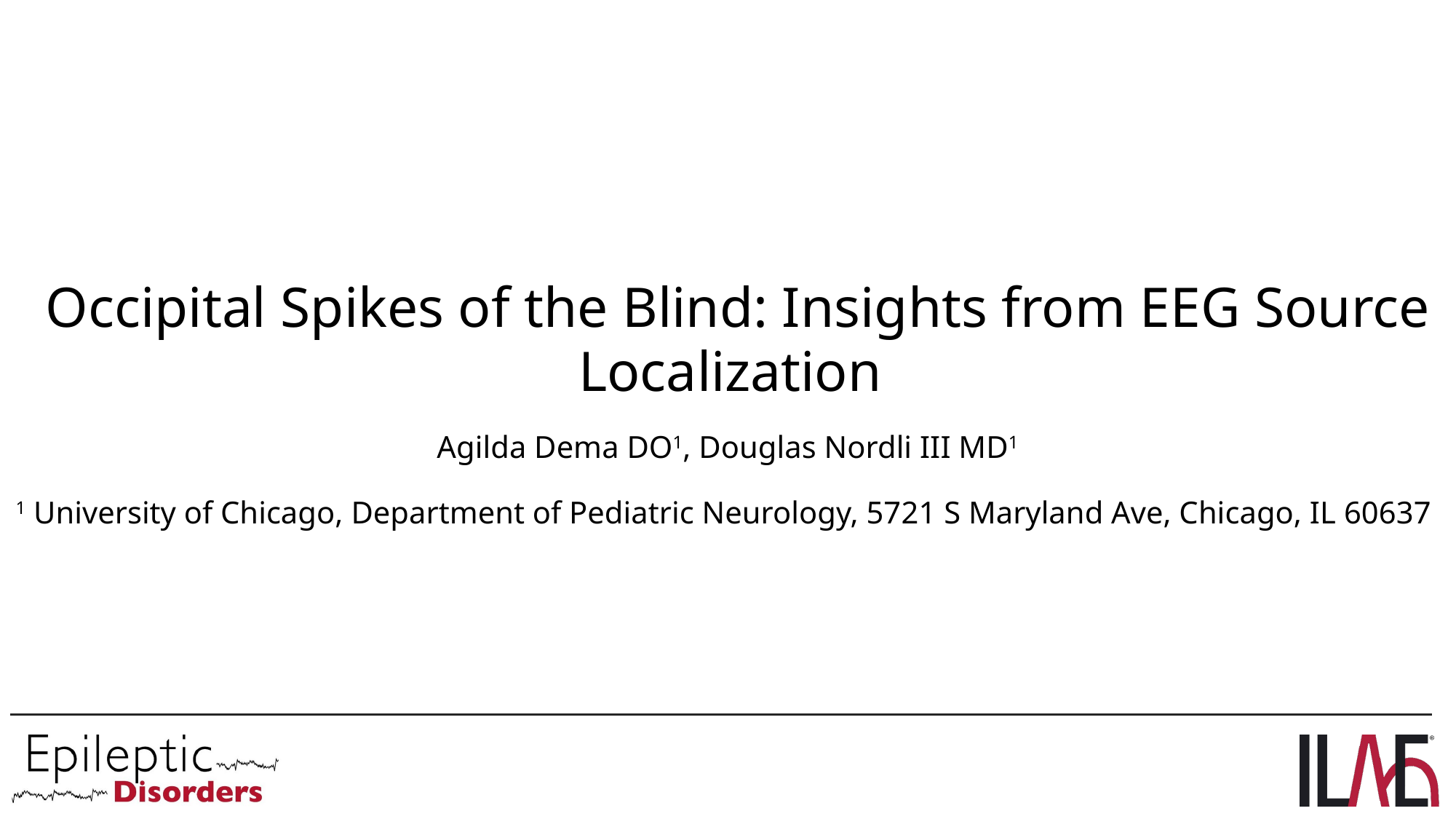

# Occipital Spikes of the Blind: Insights from EEG Source Localization
Agilda Dema DO1, Douglas Nordli III MD1
1 University of Chicago, Department of Pediatric Neurology, 5721 S Maryland Ave, Chicago, IL 60637

## Slide 2
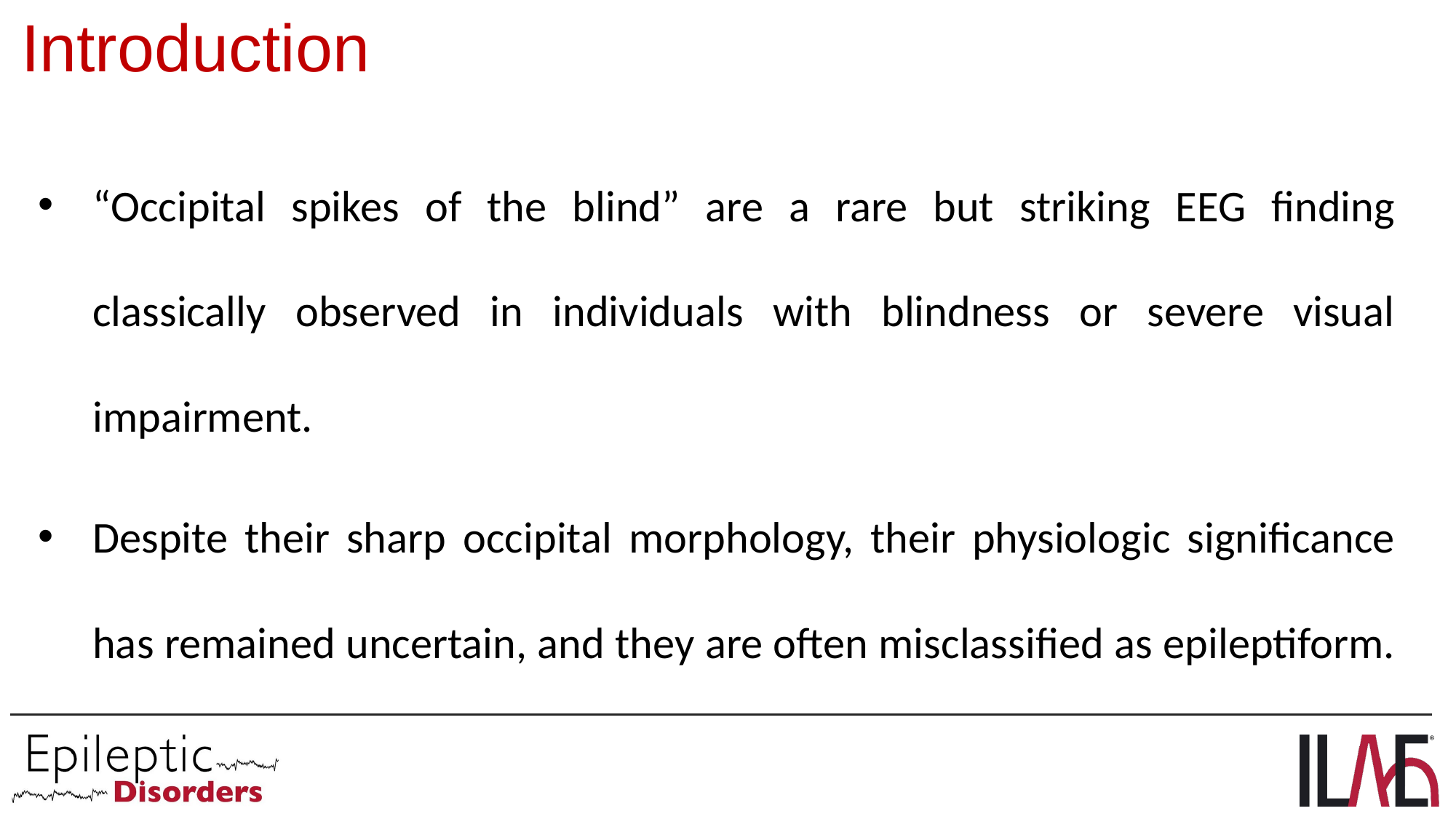

Introduction
“Occipital spikes of the blind” are a rare but striking EEG finding classically observed in individuals with blindness or severe visual impairment.
Despite their sharp occipital morphology, their physiologic significance has remained uncertain, and they are often misclassified as epileptiform.

## Slide 3
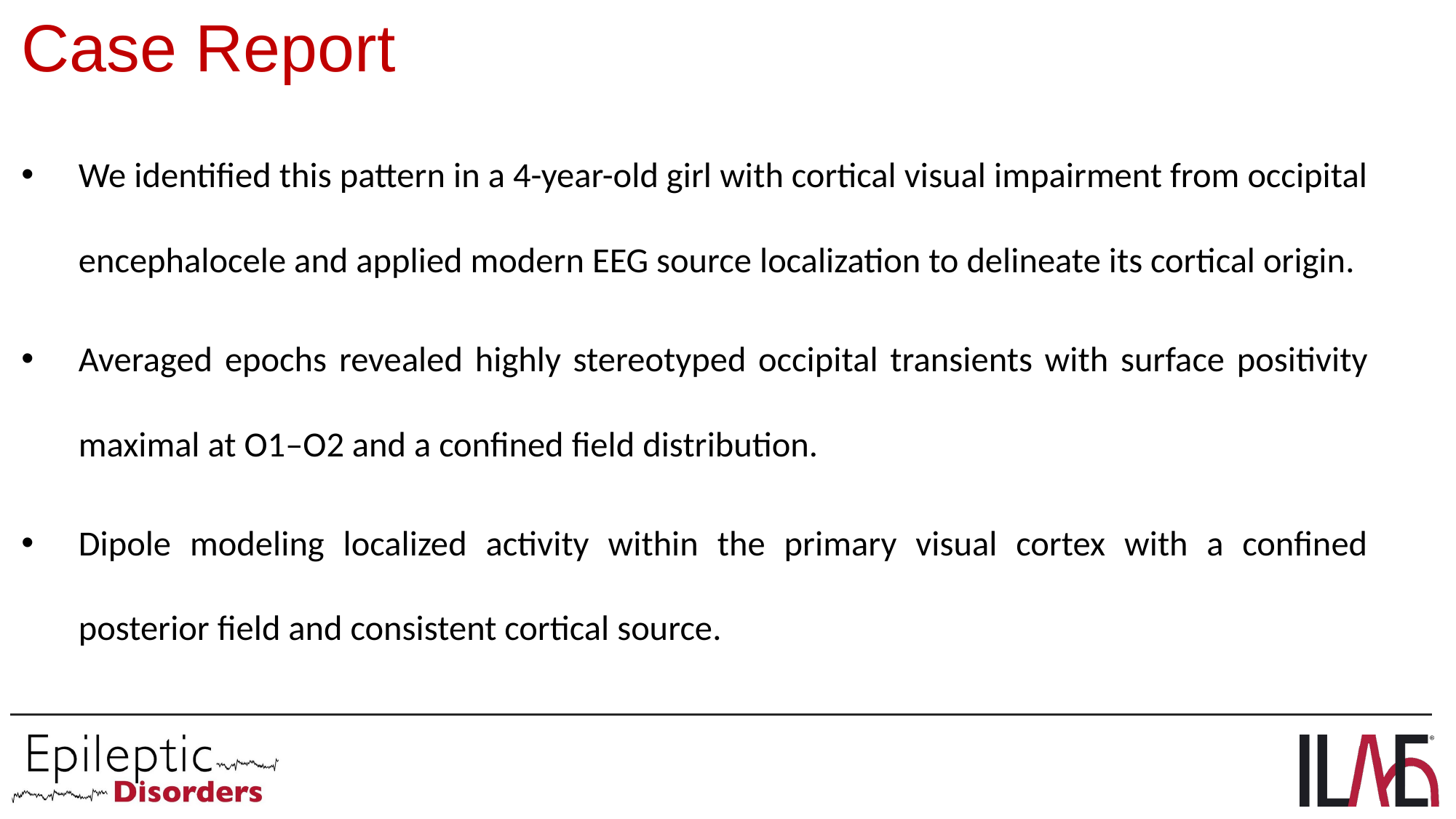

Case Report
We identified this pattern in a 4-year-old girl with cortical visual impairment from occipital encephalocele and applied modern EEG source localization to delineate its cortical origin.
Averaged epochs revealed highly stereotyped occipital transients with surface positivity maximal at O1–O2 and a confined field distribution.
Dipole modeling localized activity within the primary visual cortex with a confined posterior field and consistent cortical source.

## Slide 4
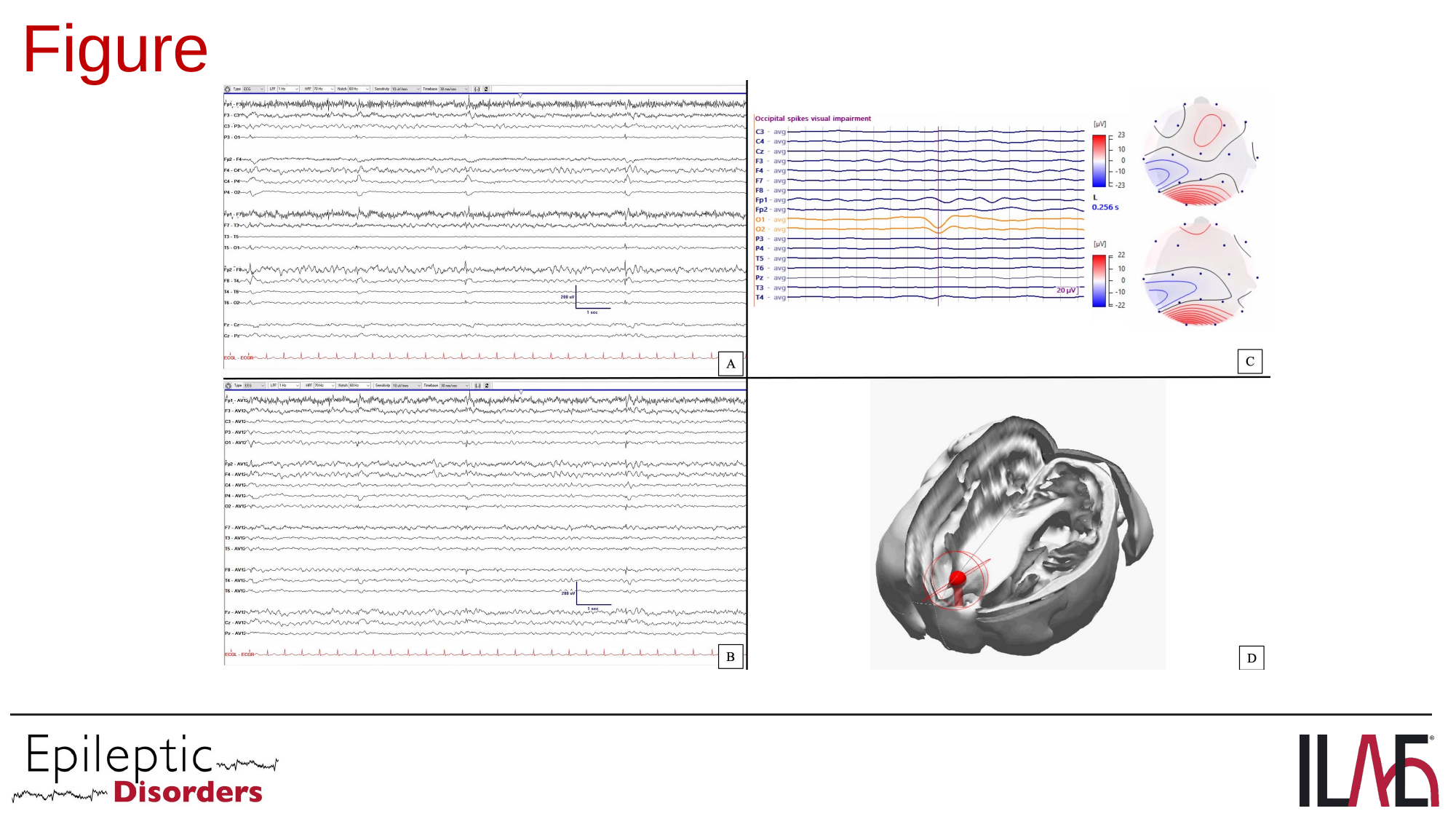

Figure

## Slide 5
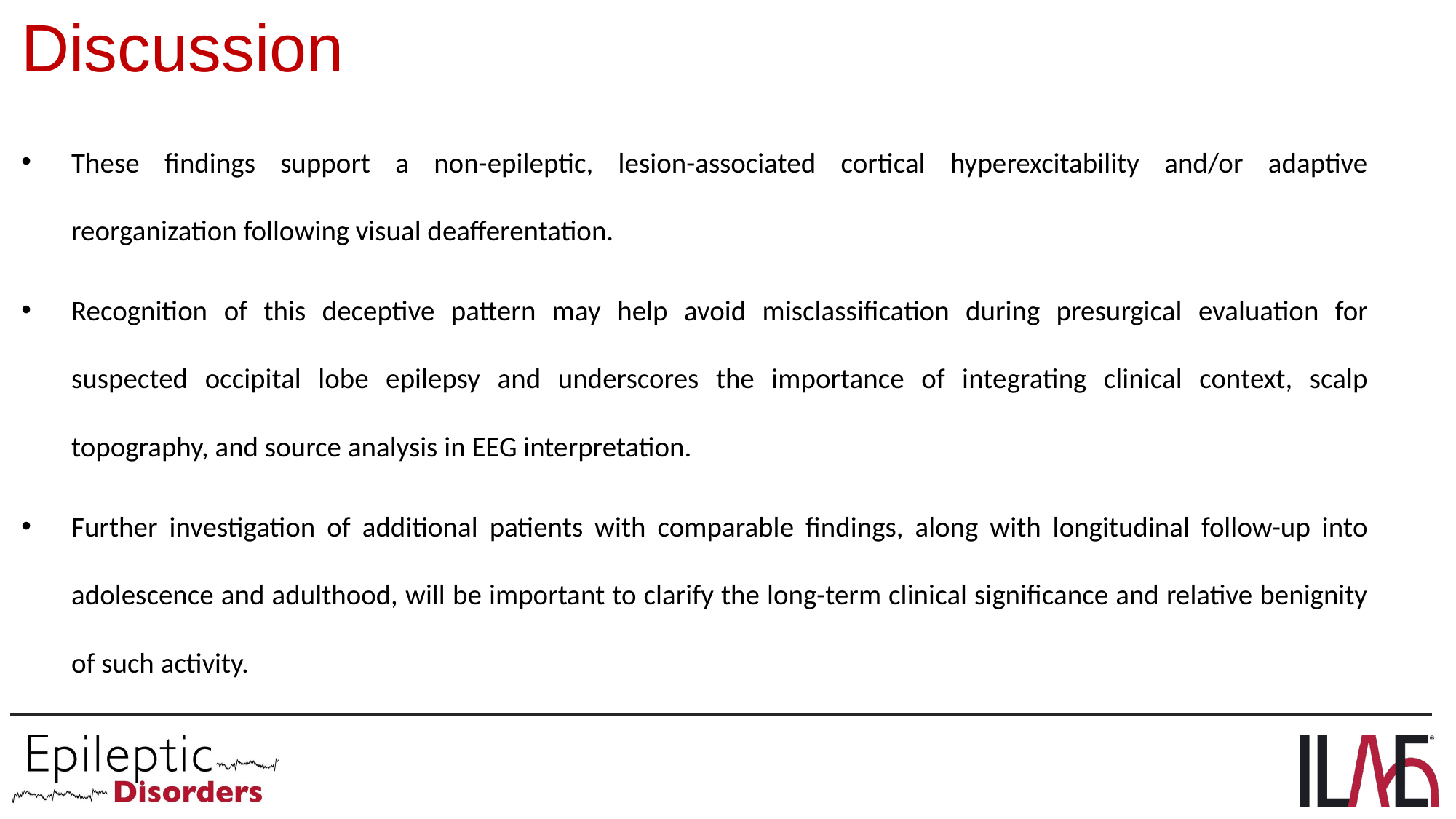

Discussion
These findings support a non-epileptic, lesion-associated cortical hyperexcitability and/or adaptive reorganization following visual deafferentation.
Recognition of this deceptive pattern may help avoid misclassification during presurgical evaluation for suspected occipital lobe epilepsy and underscores the importance of integrating clinical context, scalp topography, and source analysis in EEG interpretation.
Further investigation of additional patients with comparable findings, along with longitudinal follow-up into adolescence and adulthood, will be important to clarify the long-term clinical significance and relative benignity of such activity.

## Slide 6
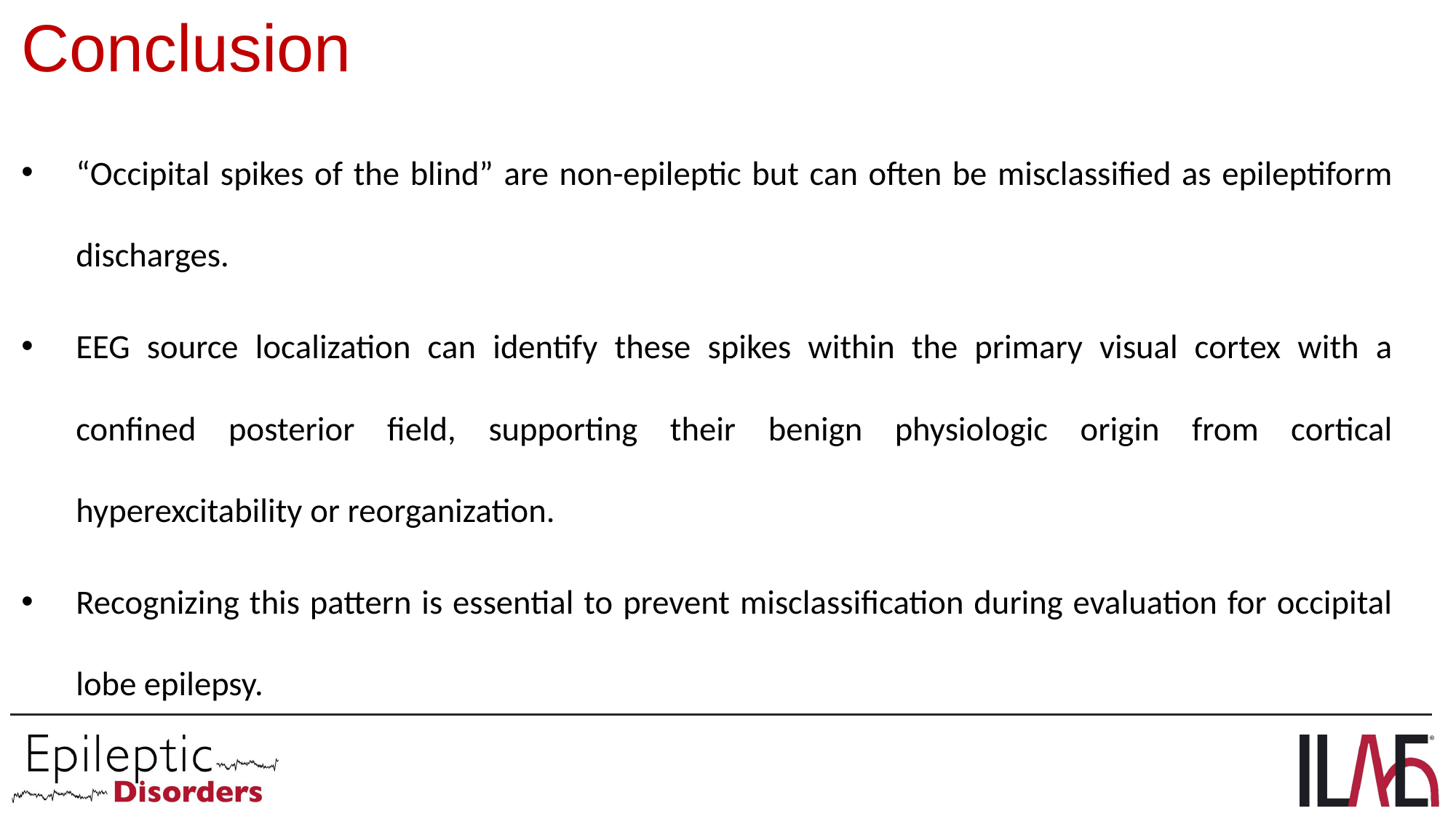

Conclusion
“Occipital spikes of the blind” are non-epileptic but can often be misclassified as epileptiform discharges.
EEG source localization can identify these spikes within the primary visual cortex with a confined posterior field, supporting their benign physiologic origin from cortical hyperexcitability or reorganization.
Recognizing this pattern is essential to prevent misclassification during evaluation for occipital lobe epilepsy.
