# Supplementary material for: Occipital spikes of the blind: Insights from EEG source localization
Source: Epileptic Disord. 2026 Mar 24;28(3):930–2. doi: 10.1002/epd2.70231 (PMC13276702; doi:10.1002/epd2.70231)
Supplement: Supplementary file 2 — Data S2: [file EPD2-28-930-s002.docx]

Test Yourself

1. Which of the following best describes the cortical source of “occipital spikes of the blind”?

1. Mesial temporal cortex
2. Parietal association cortex
3. Primary visual (calcarine) cortex
4. Frontal eye fields

Answer: C

**2. Which EEG characteristic most helps distinguish “occipital spikes of the blind” from epileptiform occipital spikes?**

1. Sharp morphology
2. Surface positivity maximal at O1–O2
3. Source localization to primary visual cortex with a confined posterior field
4. Increased frequency during sleep

**Answer:** C

3. What is a likely underlying mechanism for occipital spikes of the blind?

1. Deafferentation-related cortical hyperexcitability in visual cortex
2. Secondary epileptogenesis from temporal lobe foci
3. Retinal-originated photic afterdischarges
4. Artifact from eye movement

Answer: A
